# Management of Prolonged Ischemic Priapism: A Case Series and Critical Analysis of Early Penile Prosthesis Implantation

**DOI:** 10.1155/criu/4257471

**Published:** 2026-02-22

**Authors:** Juan Eduardo Rios Rodriguez, Déborah Cristina Andrade Neves, Alexandre Gilberto Silva, Paulo Afonso Lopes Lange, Felipe Nogueira Clementoni, Alexandre Kapteinat Lima, Bárbara Loeser Faro, Luciano Carneiro Stunitz, Marcelo Alves Aranha, Ruimário Machado Coelho, Gustavo Marquesine Paul

**Affiliations:** ^1^ Urology Residency at Hospital de Clínicas, Universidade Federal do Paraná (UFPR), Curitiba, Brazil, ufpr.br; ^2^ General Surgery Residency at Hospital de Clínicas, Universidade Federal do Paraná (UFPR), Curitiba, Brazil, ufpr.br; ^3^ Urology Department at Hospital São Marcelino Champagnat, Curitiba, Brazil; ^4^ Urology Department at Hospital de Clínicas, Universidade Federal do Paraná (UFPR), Curitiba, Brazil, ufpr.br

## Abstract

**Introduction:**

Priapism is a condition characterized by prolonged erections lasting longer than 4 h, classified as ischemic and nonischemic. This case series is aimed at reporting the therapeutic approaches employed and the outcomes observed in patients with prolonged priapism.

**Cases Description:**

We reported seven consecutive cases of prolonged priapism managed by the authors. Collected data included duration of priapism, interventions performed, and posttreatment outcomes. In Cases 1 and 2, drainage procedures and shunts were ineffective, resulting in erectile dysfunction. Cases 3 and 4 involved the implantation of semirigid prostheses after 6 and 40 days. In Case 5, an infectious complication occurred following prosthesis implantation in a patient with previous distal shunts. Case 6 demonstrated a patient with priapism had success with early prosthesis implantation. Case 7 described a patient with cocaine‐induced priapism received a prosthesis on the sixth day.

**Discussion:**

The management of ischemic priapism directly depends on the time elapsed since the onset of symptoms. After 48 h, necrosis of the corpora cavernosa becomes inevitable, and surgical shunt procedures show limited efficacy. Early implantation of penile prostheses is an effective strategy to avoid late complications such as penile fibrosis. However, the decision must consider risks such as infection and prosthesis extrusion. Shunt procedures prior to penile implantation should be indicated with caution.

**Conclusion:**

The implantation of penile prostheses is a safe and effective therapeutic option for prolonged priapism. Excessive surgical manipulation of the penis with attempts at cavernous shunting in these cases often provides no benefit and may increase the risk of prosthesis infectious complications.

## 1. Introduction

Priapism is defined as a persistent penile erection lasting more than 4 h, unrelated to sexual stimulation. Its reported incidence is approximately 1.5 per 100,000 men per year [1]. It is classified according to its etiologies as ischemic and nonischemic. The nonischemic form is related to anatomical alterations, either due to trauma or other causes, that increase blood flow, causing prolonged erection. The ischemic cause, on the other hand, is associated with a venous drainage obstruction caused by the patient′s blood factors or even as an idiopathic event, leading to severe hypoxia of the corpora cavernosa [2, 3].

The choice of treatment and its success depend on the duration of the condition. Episodes lasting less than 24 h have a good prognosis both in terms of detumescence and erectile function if managed with puncture of the corpora cavernosa, drainage, or surgical treatments based on shunts [4]. However, after 48 hours, the likelihood of success with less invasive approaches decreases, with almost 90% of erectile dysfunction after resolution of the condition [5]. The implantation of a prosthesis is a viable option for maintaining erection and improving the priapism condition, but there is still debate regarding the timing and associated complications of its placement [6].

The present study is aimed at reporting cases of long‐duration priapism, their surgical treatments, and outcomes regarding erection, as well as to discuss the treatment of late priapism. This study was approved by the Research Ethics Committee of the Complexo Hospital de Clínicas da Universidade Federal do Paraná (CAAE: 82711024.0.0000.0096).

## 2. Materials and Methods

This retrospective observational study included patients diagnosed with ischemic priapism who were admitted to the unit for a duration exceeding 24 h. Patients with incomplete medical records, patients with priapism duration lasting less than 24 h, or those diagnosed with nonischemic priapism or another pathology were excluded from the analysis. All patients in this series presented with prolonged ischemic priapism, defined clinically as very delayed presentation exceeding 48 h, although several patients arrived even earlier in the 36–48‐h interval. Given that treatment decisions were guided by the established physiologic threshold of approximately 48 h for irreversible cavernosal damage, all cases were managed according to prolonged‐ischemia protocols.

Data were collected from patients who underwent treatment for prolonged priapism, analyzing variables such as duration of clinical presentation, type of initial drainage or absence of drainage, surgical outcomes (in cases of penile prosthesis implantation), and postoperative erectile function. Given the small sample size, statistical analysis was performed using mean calculations for epidemiological comparison. Below, we describe the collected cases along with their outcomes. This case was structured in accordance with the CARE guidelines for case reports [7].

## 3. Results

### 3.1. Case 1

**Figure 1:**
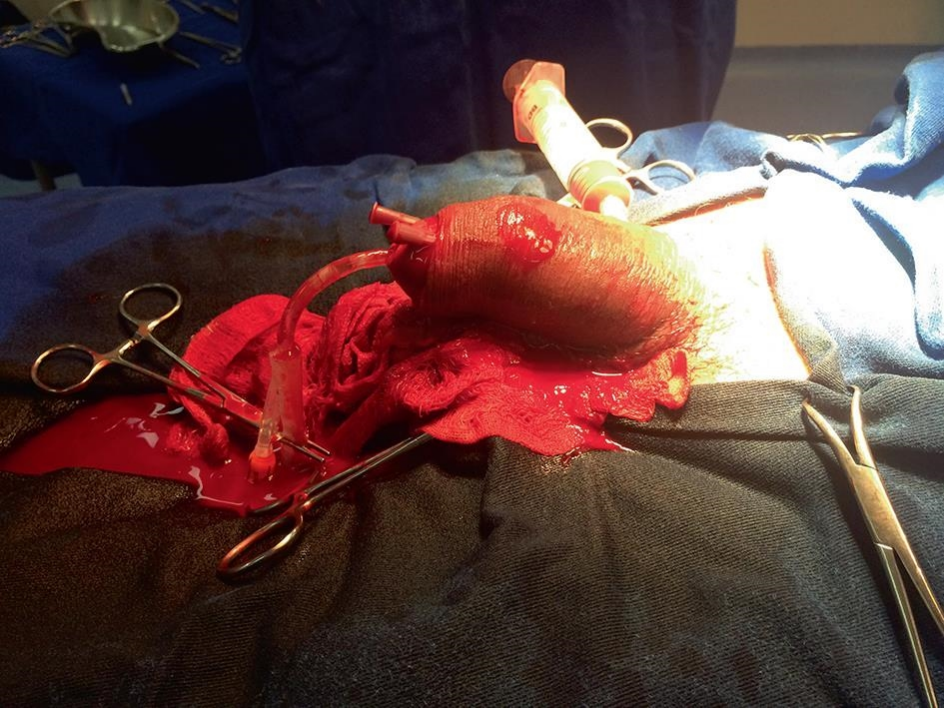
Patient Number 1 during winter procedure in attempted treatment for priapism.

A 35‐year‐old man presented with priapism lasting 40 h, without any prior corporal drainage before admission. Cavernosal blood aspiration confirmed ischemic priapism. Cavernous body evacuation and washing were performed without success. Winter, Al‐Ghorab, and Quackels procedures were attempted, all without satisfactory response, and the erection persisted (Figure 1). It was decided to maintain the erection without further procedures, and the patient was discharged after pain control. After 3 weeks, there was complete improvement in erection, but progression to erectile dysfunction without response to an optimized dose of phosphodiesterase 5 inhibitor. At the 6‐month follow‐up, the patient showed no response to either oral therapy or intracavernosal injection.

### 3.2. Case 2

A 58‐year‐old man presented with priapism lasting 45 h. Ischemic priapism was confirmed by cavernosal blood gas analysis. Initial drainage and cavernous body washing were performed without response. Winter, Al‐Ghorab, and Burnett procedures were performed, resulting in improvement and maintenance of only a flaccid erection. The patient showed late postoperative improvement in erection but experienced erectile dysfunction refractory to oral therapy and intracavernosal injection throughout the 6‐month postoperative period.

### 3.3. Case 3

A 37‐year‐old man presented with priapism lasting 72 h. Corporal drainage and a Winter shunt procedure were performed. Ischemic priapism was confirmed by cavernosal blood gas analysis. It was decided not to perform any surgical procedure or shunt. A semirigid prosthesis was implanted, which had to be placed after 6 days due to material availability. After surgery, there was resolution of pain and satisfactory healing, with functional use of the prosthesis with good sexual quality of life and subjective satisfaction reported at the 1‐year follow‐up, without complications.

### 3.4. Case 4

A 52‐year‐old male patient, no drugs or diseases related, presented to the emergency unit with priapism lasting 96 h without prior treatment. Cavernosal blood gas analysis confirmed ischemic priapism. It was decided to perform the implantation of a semirigid penile prosthesis without any drainage technique, either by puncture or surgery. Due to material availability, the prosthesis was implanted after 40 days, but with good surgical results, pain improvement, and a functional prosthesis. Unfortunately, the patient was later diagnosed with stomach adenocarcinoma after resolution of the priapism at the 6th month, requiring chemotherapy, unrelated to the priapism episode.

### 3.5. Case 5

A 57‐year‐old male patient presented with priapism lasting 50 h. After ischemia was confirmed by cavernosal blood gas analysis, evacuation and washing of the corpora cavernosa were performed, as well as winter drainage, without success. The Al‐Ghorab procedure was attempted as a treatment, also without success. Due to the prolonged duration of the priapism, it was decided to implant a semirigid penile prosthesis. However, on the sixth postoperative day, purulent discharge from the right side of the glans was observed, requiring surgical revision, removal of the prosthesis, and intravenous antibiotic therapy. The patient also developed urinary retention, and urethral stenosis was detected. The prosthesis was permanently lost, and the patient declined further procedures during the first postoperative year.

### 3.6. Case 6

A 26‐year‐old male patient presented with priapism lasting 168 h, with no previous treatment attempts before the first evaluation. The patient had been taking 30 mg of olanzapine for clinically stable schizophrenia and had been followed by psychiatry for 5 years. He developed an erection without any traumatic or drug‐related history. Due to the prolonged priapism, it was decided to proceed with an upfront semirigid penile prosthesis implantation, without any aspiration technique, in agreement with the patient, who prioritized maintaining erectile function. Six months after the procedure, the patient remains satisfied with the surgery. Psychological and psychiatric teams provided support before and after the surgery.

### 3.7. Case 7

**Figure 2:**
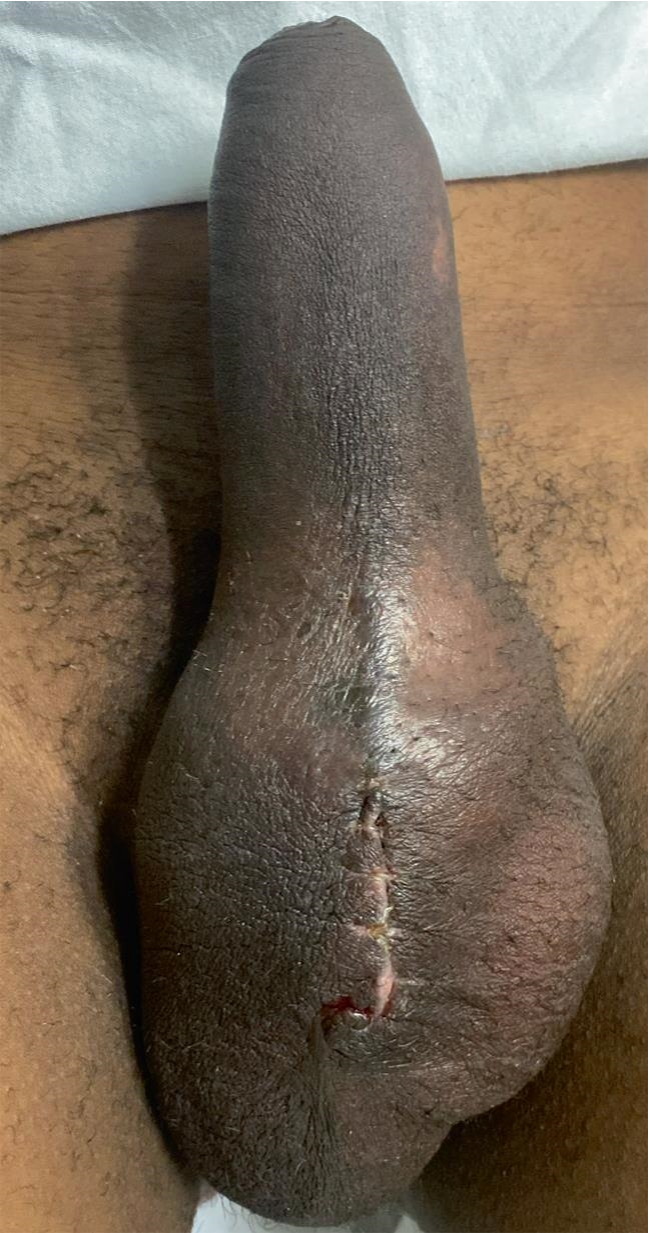
Physical examination on the seventh day after penile prosthesis surgery (Patient Number 7).

A 53‐year‐old male patient, a cocaine and chlorpromazine user, presented with 120 hours of priapism, with the main complaint being local pain, without any prior drainage before arrival at the unit. Cavernosal blood gas analysis and penile Doppler confirmed ischemic priapism. The patient agreed to proceed with an upfront semirigid prosthesis implantation. The procedure was performed on the sixth day of priapism, with pain relief on the first postoperative day. On the third day after surgery, the patient experienced intense pain, but without signs of local infection, only a slight hematoma in the scrotal sac (Figure 2). Up to the sixth month of follow‐up, the patient reported adequate satisfaction and sexual quality of life with the prosthesis.

The mean age of the patients was 45.4 years (range: 26–58), and the mean duration of priapism prior to seeking medical care was approximately 84.4 h (range: 40–168). Among the seven patients, two who did not undergo penile prosthesis implantation experienced erectile dysfunction refractory to pharmacological therapy and intracavernosal injections (28.5%). Among those who underwent prosthesis implantation, two had undergone puncture drainage procedures (winter procedure), and only one underwent the Al‐Ghorab technique, which resulted in the sole case of prosthesis extrusion (14%). Primary prosthesis implantation was performed in four patients (57.14%), all of whom demonstrated good postoperative outcomes and satisfactory adaptation to sexual activity (Table 1).

**Table 1:** Cases summary.

Case	Age	Etiology	Previous procedures	Prosthetic implant	Duration of priapism episode	Outcomes after 6 months
01	35	Idiopathic	Winter procedure, Al‐Ghorab procedure, Quackels procedure	No	40 h	Erectile dysfunction unresponsive to drug treatment or intracavernous injection
02	58	Idiopathic	Winter procedure, Al‐Ghorab procedure, Burnett procedure	No	45 h	Erectile dysfunction unresponsive to drug treatment or intracavernous injection
03	37	Idiopathic	Winter procedure	Yes (6th day of priapism)	72 h	Good response to surgery, functional prosthesis
04	52	Idiopathic	None	Yes (40th day of priapism)	96 h	Good response to surgery, functional prosthesis
05	57	Idiopathic	Winter procedure, Al‐Ghorab procedure	Yes (3rd day of priapism)	50 h	Extrusion of penile prosthesis infection and erectile dysfunction
06	26	Possible drug‐related	None	Yes (7th day of priapism)	168 h	Good response to surgery, functional prosthesis
07	53	Possible drug‐related	None	Yes (6th day of priapism)	120 h	Good response to surgery, functional prosthesis, testicular sac hematoma

## 4. Discussion

The treatment of ischemic priapism depends on the time between the onset of symptoms and the time of treatment, with its objectives being penile detumescence, pain relief, preservation of erectile function after the episode, and minimizing complications such as infection or bleeding. Due to its etiology, aspiration of the corpora cavernosa, with or without the injection of alpha‐adrenergic substances such as phenylephrine, can be helpful in cases lasting less than 24 h [1].

After 24 h, due mainly to sustained tissue hypoxia in the corpora cavernosa, a state of intense tissue ischemia is created, which can progress to necrosis as the duration increases without resolution. In these cases, aspiration and injection of alpha‐adrenergic medications alone show minimal rates of resolution, and surgical shunts are required for a better prognosis, although they present rates of 14%‐25% erectile dysfunction postoperatively [1, 8].

In more advanced cases, such as after 48 h, even decompression surgeries and shunts do not yield satisfactory success rates for the treatment of priapism, particularly when assessing erectile dysfunction in the postoperative follow‐up of these patients [9, 10]. Even patients who underwent invasive procedures have nearly a 90% chance of erectile dysfunction that is difficult to treat [5]. All reported cases treated with only decompressive maneuvers or shunts developed severe erectile dysfunction unresponsive to oral medication or injectable treatments. This is consistent with current European guideline interpretations, which emphasize that irreversible smooth muscle injury becomes likely after approximately 48 h of ischemia and that, in delayed presentations, functional recovery is extremely limited. For this reason, contemporary recommendations suggest that early consideration of penile prosthesis implantation is reasonable in prolonged or refractory ischemic priapism, aiming to avoid the technical challenges and higher complication rates associated with delayed surgery [11].

This difficulty in maintaining erectile function in cases of late priapism is due to prolonged ischemia and necrosis of the corpora cavernosa after 48 h [12]. Even if decompression and resolution of the erection occur, fibrosis will form in the necrotic tissue, resulting in rigid tissue across the entire length of the penis, making a satisfactory erection impossible [9].

A viable surgical option is the implantation of a penile prosthesis, either malleable or inflatable. A prosthesis is an option for cases of priapism that do not respond to surgical treatment, whether with surgical shunts or other decompression alternatives for the corpora cavernosa [13]. In such cases, it is usually recommended to wait for fibrosis and healing of surgical procedures before evaluating the placement of a definitive prosthesis, but this can also cause penile shortening due to the same fibrotic reaction [4].

However, there is debate regarding the immediate placement of a penile prosthesis in cases where the initial evaluation indicates a duration of more than 48 h. This option is based on the minimal success rates of phenylephrine injections or aspiration regarding the maintenance of unsatisfactory erections when surgical treatments without a prosthesis are performed, especially when there is evidence of necrosis of the corpora cavernosa [14].

To aid in the decision of prosthesis implantation, the use of methods such as magnetic resonance imaging (MRI) and penile ultrasound is more readily available today. Penile Doppler ultrasound has good accuracy in assessing blood flow in the cavernous artery; however, MRI is superior in evaluating ischemia and probable necrosis, showing nearly 100% correlation between imaging findings and histopathology in one study [15]. Frozen biopsy during surgical decompression or before prosthesis implantation confirms tissue necrosis, and it is recommended.

Psychological evaluation of the patient is crucial, as they may experience some level of psychological distress when informed about the condition and its prognosis, as well as the possibility of prosthesis use. Psychological support is important and decisive, as is clear communication and understanding from the surgical team. The possibility of using an inflatable or malleable prosthesis should be discussed with the patient, although the malleable prosthesis is more readily available and can be used as a bridge treatment if the patient wishes to switch to an inflatable one in the future [16].

Regarding the timing of prosthesis implantation, two factors should be considered: postoperative infection and technical difficulty. Studies evaluating priapism cases treated with prosthetic intervention raise concerns about the number of infectious complications when the prosthesis is placed immediately or early. These complications are also related to previous treatments the patient underwent, which could impact the likelihood of infections and even prosthesis extrusion [1, 17]. However, in patients with priapism lasting more than 48 h, if there is the possibility and availability of penile prosthesis implantation, distal shunts or other procedures should not be performed, as even the more aggressive ones do not yield good outcomes in terms of erectile function prognosis. These procedures can also delay prosthesis implantation and increase the risk of complications, such as extrusion, as distal shunts can facilitate prosthesis extrusion even after suturing, as occurred in one of the reported cases [18].

Technical difficulty can also be a factor indicating better results when performing an earlier prosthesis implantation. If we wait 3 months for implantation, the cavernous tissue will have intense fibrosis, making the surgery more challenging, with a higher risk of injury to the corpora cavernosa and even the need for a cavernosotomy. If performed at the time of priapism, the implant is technically simpler and may result in fewer complications [6].

There are alternatives for treating late priapism. Penoscrotal decompression has emerged in some recent studies as a promising method, but the postoperative erectile function outcomes are conflicting between studies, with one reporting almost 90% erectile function and another reporting only 15% [11, 19]. It should also be noted that conducting a prospective study on priapism is challenging, as most of these studies are retrospective with limited patient epidemiological data and long‐term outcomes

The limitations of this study are inherent to its design, as it is a retrospective study with a small sample size, given that priapism—particularly in prolonged cases—is rare. An important limitation of this study is the absence of standardized and validated instruments to assess postoperative sexual function, such as IIEF‐5 or SHIM. Follow‐up was based on patient‐reported satisfaction and subjective functional assessment. Further studies with larger patient cohorts and a prospective design are needed.

## 5. Conclusions

The treatment of late ischemic priapism is challenging. Decompression and intracavernosal injections show limited results in treating cases after 48 hours and have high rates of erectile dysfunction. Penile prosthesis implantation, whether malleable or inflatable, is a viable option in these cases, as if tissue necrosis is confirmed or even suspected, erectile dysfunction can already be treated safely with implantation. If prosthesis implantation is an option from the initial evaluation, care should be taken not to perform surgical procedures such as distal shunts or drainage, as they can increase the risk of infection or even prosthesis extrusion. Multidisciplinary patient follow‐up, especially with psychological support, is always essential.

## Funding

No funding was received for this manuscript.

## Disclosure

All authors have read and approved the final version of the manuscript, Juan Eduardo Rios Rodriguez had full access to all of the data in this study and takes complete responsibility for the integrity of the data and the accuracy of the data analysis.

## Ethics Statement

This study was approved by the Research Ethics Committee of the Complexo Hospital de Clínicas da Universidade Federal do Paraná (CAAE: 82711024.0.0000.0096). The participants provided written, informed consent.

## Conflicts of Interest

The authors declare no conflicts of interest.

## Data Availability

The data that support the findings of this study are available from the corresponding author upon reasonable request.
